# The Effect of Pr Doping Contents on the Structural, Microstructure and Dielectric Properties of BaBi_2_Nb_2_O_9_ Aurivillius Ceramics

**DOI:** 10.3390/ma15165790

**Published:** 2022-08-22

**Authors:** Michał Rerak, Jolanta Makowska, Katarzyna Osińska, Tomasz Goryczka, Anna Zawada, Małgorzata Adamczyk-Habrajska

**Affiliations:** 1 Faculty of Science and Technology, University of Silesia, 1A 75 Pułku Piechoty St., 41-500 Chorzów, Poland; 2 Faculty of Production Engineering and Materials Technology, Czestochowa University of Technology, Aleja Armii Krajowej 19 Str., 42-200 Czestochowa, Poland

**Keywords:** ceramics, Aurivillius structure, BaBi_2_Nb_2_O_9_, praseodymium Pr^3+^

## Abstract

Aurivillius BaBi_2_Nb_2_O_9_ and Ba_1-x_Pr_x_Bi_2_Nb_2_O_9_ ceramics were successfully synthesized by a simple solid state reaction method. Ceramics were prepared from reactants: Nb_2_O_5_, Bi_2_O_3_, BaCO_3_ and Pr_2_O_3_. The microstructure, structure, chemical composition, and dielectric properties of the obtained materials were examined. Dielectric properties were investigated in a wide range of temperatures (T = 20–500 °C) and frequencies (f = 0.1 kHz–1 MHz). The obtained ceramic materials belong to the group of layered perovskites, crystallizing in a tetragonal structure with the space group I4/mmm. Modification of the barium niobate compound with praseodymium ions influenced its dielectric properties and introducing a small concentration of the dopant ion causes a slight increase in the value of electric permittivity and shifts its maximum towards higher temperatures.

## 1. Introduction

As an important kind of functional material, perovskite ceramics, e.g., PbZrO_3_ –PbTiO_3_ [1,2] or PbFe_1/2_Nb_1/2_O_3_ [3,4], are desirable for any applications operating in the automotive, aerospace, and bio-medical industries. However, their preparation, application, and disposal have caused serious environmental pollution connected with the toxicity of lead. Alternative candidates which have attracted significant attention are the Aurivillius compounds described by the general *Formula (1)* [5,6,7]:

(1) $${\left(M_{2}O_{2}\right)}^{2+}{\left(A_{n-1}B_{n}O_{3m+1}\right)}^{2-}$$

The presented chemical formula shows that these structures are composed of regularly arranged layers (Bi_2_O_2_)^2+^ and perovskite blocks (A_n−1_B_n_O_3m+1_)^2−^ where n denotes, the number of layers of these blocks [8,9]. The A subnetwork is usually occupied by ions of elements such as: Bi^3+^, Sr^2+^, Ba^2+^, Ca^2+^ [10,11], while the B subnetwork has its place for Nb^5+^, Ta^5+^, W^6+^, Mo^6+^ [11,12,13]. The place of the M cation in the bismuth-oxygen layers is usually occupied by bismuth ions [5], but also partially by other ions, such as Gd^3+^ or Ce^3+^ [14]. The numerous possibilities of ion substitutions, both in the structure of perovskite blocks and in bismuth-oxygen layers, create a broad spectrum of modifications of the discussed compounds and thus almost any modification of their physical properties [5].

BaBi_2_Nb_2_O_9_ (BBN) ceramics is a polycrystalline material belonging to the family of layered perovskites. This material is characterized by the layered structure of Aurivillius, which is made up of perovskite layers cut from a regular crystal lattice by two planes (001) intertwined with bismuth-oxygen layers. Adjacent pseudo-perovskite layers are shifted in relation to each other in the family of directions [110]. Thus, in the said direction, sections of chains composed of oxygen octahedrons connected with each other by their apexes successively follow one another, as well as sections of regular polyhedrons BO_12_ and AO_12_ connected by edges. BBN ceramics can crystallize in a rhombic or tetragonal structure [15,16,17].

Since the ceramics of barium bismuth niobate is a material that is still subject to intensive research, various research groups are still looking for the optimal technology and modifiers that can significantly improve the parameters already obtained to generate new, completely different properties in the material [18,19,20]. One such promising admixture is praseodymium.

The aim of this study was to synthesize and fabricate BBN and Ba_1-x_Pr_x_Bi_2_Nb_2_O_9_ (BPBN) for x = 0; 0.2, 0.4, 0.6, 0.8, 0.1 ceramics by solid state reaction. Then, the morphology, crystalline structure and dielectric properties of the obtained materials were studied. Expanding the knowledge of structural and dielectric properties will create the opportunity to design innovative functional materials in the future.

## 2. Materials and Methods

Barium bismuth niobate (BaBi_2_Nb_2_O_9_) was doped in the A subnetwork with praseodymium ions (Pr^3+^) for mole fractions in the range x = 0.01–0.1. The reactants used were niobium oxide (V) Nb_2_O_5_ (Aldrich 99.9%, St. Louis, MO, USA), bismuth oxide (III) Bi_2_O_3_ (Aldrich 99.9%), barium (III) carbonate BaCO_3_ (Aldrich 99.9%) and praseodymium oxide (III) Pr_2_O_3_ (POCH, CZDA).

The test material was produced using conventional technology, i.e., solid-phase synthesis by free sintering in air.

The stoichiometric mixture of oxides and carbonates was grinding in a planetary ball mill for t = 24 h (with 97% ethyl alcohol, POCH CZDA) at 200 revolutions per minute. After milling, the powders were subjected to drying. Then, the dried mixture of powders was compacted into pellets of d = 25 mm in diameter by pressing under pressure of *p* = 300 MPa in a stainless- steel die. The synthesis was carried out at T = 950 °C in corundum crucible with air atmosphere for t = 4 h. After the thermal treatment, the pellets were crushed in a mortar and the synthesized material was subjected to wet milling and drying again. Before sintering, the compacts were formed in a stainless-steel die of d = 10 mm in diameter and pressed into pellets under the pressure of *p* = 600 MPa. The sintering was carried out in ambient air at temperature T = 1100 °C for t = 2 h. 

The Archimedes method with distilled water was employed to evaluate the sample density. 

In order to determine the thermochemical properties of obtained powders, differential thermal analysis (DTA) and thermogravimetric analysis (TG/DTG) were used. Thermal analysis of the produced material was carried out using the MOM q1500D derivatograph (Budapest, Hungary), Paulik-Paulik-Erdey system.

The microstructure and chemical composition of the final ceramics were examined with a scanning electron microscope (SEM) JSM-7100F (JEOL, Tokyo, Japan) equipped with an energy dispersive spectrometer (EDS) NORAN Vantage (Thermo Fisher, Waltham, MA, USA). The procedure of registering sample images was based on the random selection of several fields distributed over the entire surface of the tested ceramics.

The phase analysis and structure details of the tested material were performed based on the X″Pert PRO X-ray diffractometer by PANAlytical, Malvern, UK, (Cu-K_α_ radiation). The X-ray diffraction patterns were measured at room temperature in an angular 2θ range: 10–140°. The step-scan mode was applied with step 0.04° and time adjusted to receive accurate counting statistics. The intensities and positions of the diffraction lines of the experimental diffraction pattern were compared with that of the international ICDD-PDF4 database (JCPDS card no. 00-40-0355). The structure refinements were performed by Rietveld’s method [21] using the LHPM computer program.

The computerized automatic system based on precision LCR meter Agilent, Santa Clara, CA, USA, E4980A was used to measure the temperature dependencies of permittivity in a frequency range f = 0.1 kHz–1 MHz.

## 3. Results

**Figure 1:**
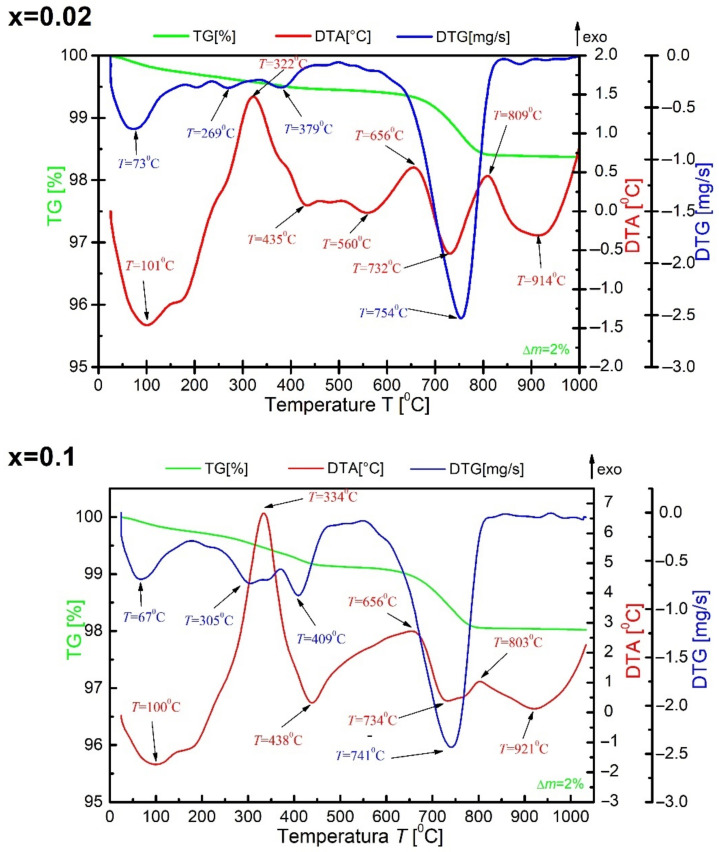
The results of the thermal analysis of the stoichiometric mixture of starting oxides and carbonates of BaBi_2_Nb_2_O_9_ ceramics modified with praseodymium ions for x = 0.02 and 0.1.

A set of thermal analysis methods (DTA, TG, DTG) was used to determine the thermal effects occurring in the tested material before synthesis. The measurement was performed in a range from room temperature to T = 1050 °C at the sample heating rate of υ = 10 °C/min. For all ceramic materials doped with praseodymium, the nature of the obtained DTA, DTG and TG curves is similar (as evidenced by Figure 1—for x = 0.02 and x = 0.1), the relationships of DTA, DTG and TG for the ceramic material modified with praseodymium ions are discussed for x = 0.02.

It can be noticed that weight loss takes place in three distinct stages. The first weight loss can be observed from room temperature to T = 250 °C. It corresponds to the minimum on the DTG curve at T = 73 °C and the endothermic maximum at T = 101 °C on the DTA curve. This loss can be attributed to the evaporation of the sample moisture. The second weight loss is observed in the temperature range from T = 250 °C to T = 660 °C. The minima on the DTG curve at the temperatures T = 269 °C and T = 379 °C correspond to it. This loss is also accompanied by exothermic maxima at T = 322 °C and T = 656 °C and endothermic maxima at T = 435 °C and T = 560 °C on the DTA curve. The second weight loss results from the transition of the bismuth oxide phase from the α phase to the β phase. The third weight loss was recorded for the temperature range from T = 650 °C to T = 830 °C. A large minimum accompanies it on the DTG curve at the temperature of T = 754 °C. Moreover, two maxima appear on the DTA curve in the discussed temperature range. The endothermic maximum is marked on the aforementioned curve at T = 732 °C. On the other hand, the exothermic maximum appears at the temperature of T = 809 °C. The discussed weight loss is related to the thermal decomposition of barium carbonate and the release of carbon dioxide CO_2_ and slight evaporation of bismuth oxide (III). The total weight loss is Δm = 2%. When discussing the thermal analysis results, one should also mention the additional endothermic maximum observed on the DTA curve at the temperature T = 914 °C, which is related to the nucleation of the new BBN phase. Above the temperature T = 950 °C, no thermal effects were observed in the sample.

**Figure 2:**
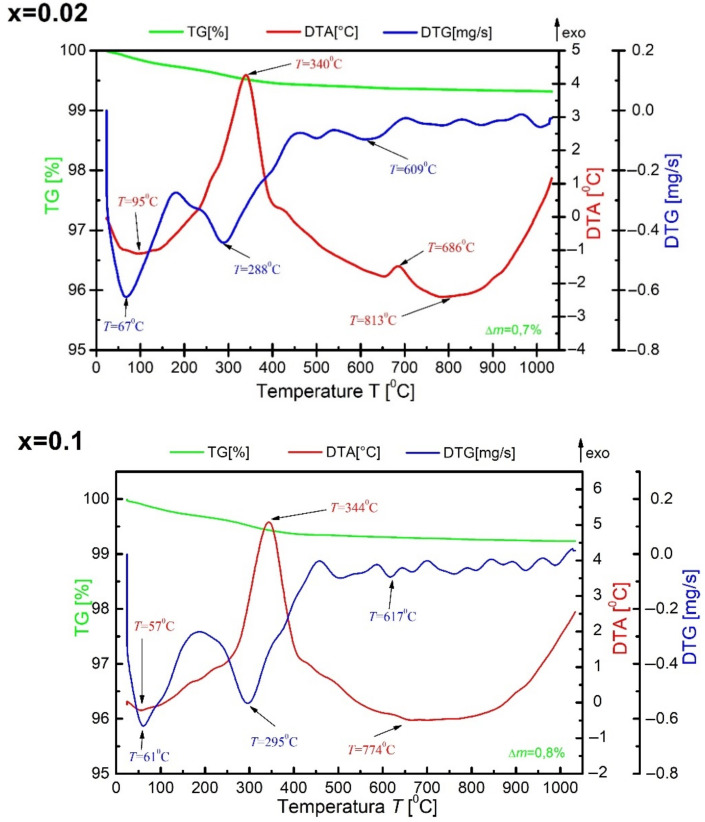
Results of thermal analysis of BaBi_2_Nb_2_O_9_ material modified with praseodymium ions after synthesis for x = 0.02 and x = 0.1.

Thermal analysis was also performed for BBN ceramic powder doped with praseodymium after synthesis (Figure 2).

The analysis of the TG curve shows that the weight loss in the case of synthesized powders is slow and small—the total weight loss is only Δm = 0.7%. There are also four characteristic DTA maxima at the temperatures T = 95 °C, T = 340 °C, T = 686 °C and T = 813 °C. The last three probably correspond to the changes in the crystal structure of tested sample, manifested, among other things, in the movement of oxygen octahedrons, which is widely described in more detail in paper [22]. Moreover, slight maximum is visible on DTA and also DTG curves around temperature 269 °C. The maximum seems to confirm our earlier theories about crystallographic structure evolution in this temperature range [22]. It is commonly known that the ions exchange their positions –the barium ions enter into bismuth oxide layers and replace the bismuth ions. The bismuth ions occupy, in turn, the free space in the perovskite blocks [23,24]. In the final effect, the “average” macroscopic structure is tetragonal. Still, this structure contains the polar nano-regions (clusters) with orthorhombic distortion, which cause the relaxor-type ferroelectric properties observed in the material. The mentioned clusters start gradually changing their symmetry from orthorhombic to tetragonal at sufficiently high temperatures, which leaves a mark on the DTA and DTG curves. The fastest course of weight loss was recorded at T = 67 °C. It is a characteristic point of most measurements made with this method and corresponds to moisture loss. Above the temperature T = 820 °C, no thermal effects were registered. This information allows us to correctly determine the temperature of the synthesis, which should be higher than all thermal processes.

**Figure 3:**
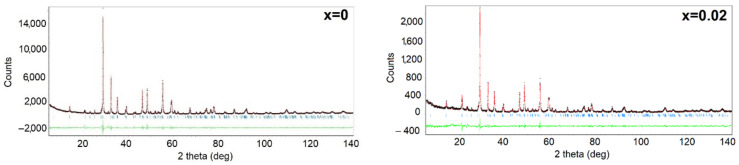

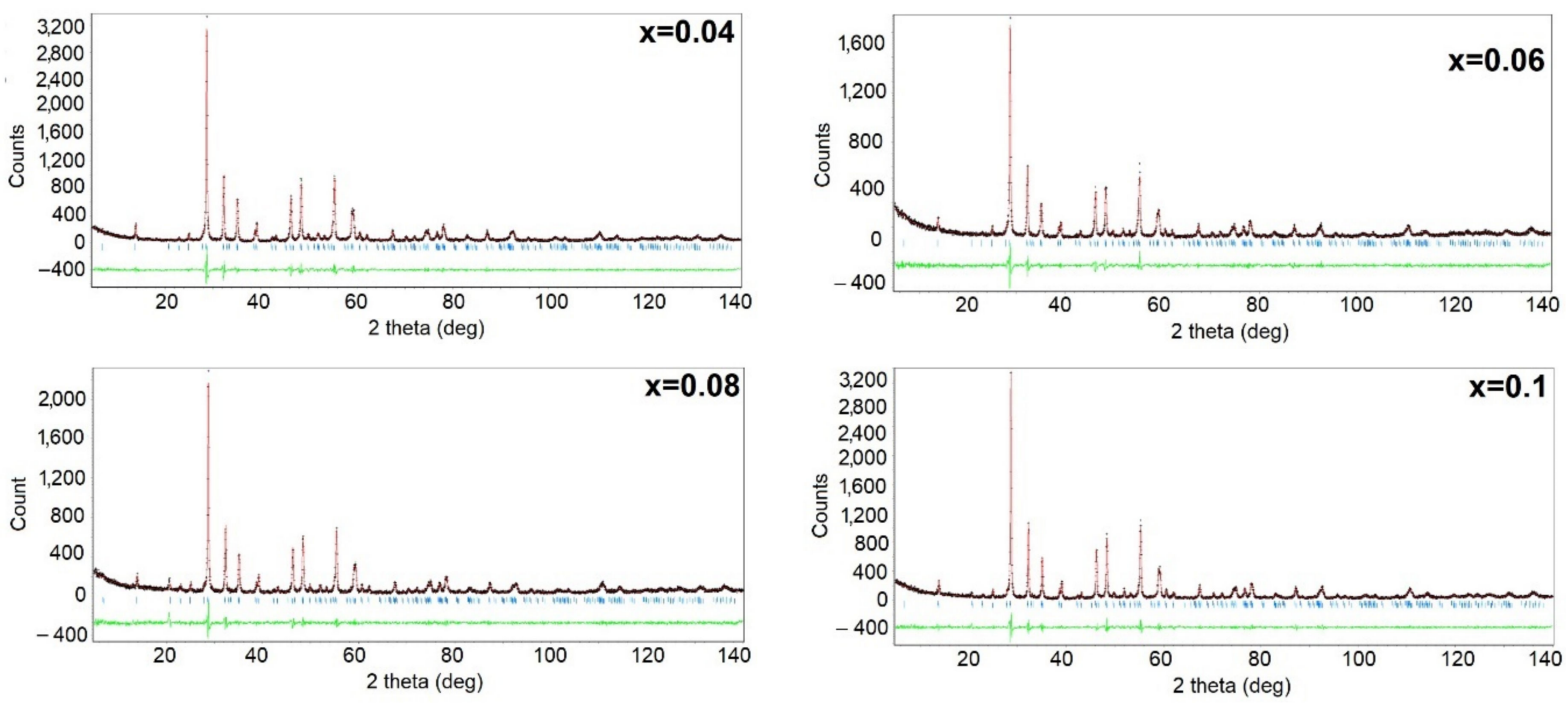
Results of matching the X-ray spectrum of BBN ceramics doped with Pr^3+^ ions.

In order to determine the effect of the modifier concentration on the crystal structure of BBN ceramics, an X-ray diffraction test was performed. The first step in analyzing the obtained results was phase identification which consisted of comparing the intensity and position of the diffraction lines of the experimental diffraction pattern with the pattern from the international database. The analysis allowed us to state that the crystalline grains of the discussed ceramic materials are single-phase at room temperature. All X-ray diffraction patterns revealed diffraction lines belonging to the barium bismuth niobate (BaBi_2_Nb_2_O_9_) tetragonal phase—JSPDS card no 00-040-0355. Modifying the material with praseodymium ions for mole fractions x = 0.02–0.10 does not change the crystal structure of the compound, the symmetry of which can be described using the I4/mmm space group. In order to receive information from praseodymium ions addition impact on crystal structure, the lattice parameters were determined using the Rietveld method [25,26,27]. The crystallographic data from the JSPDS card 00-040-355 were used as a starting model for the refinement. Results of the refinement are shown in Figure 3, whereas calculated values of the crystallographic parameters as well as the reliability factors determining the goodness of refinement (R_p_-reliability factor of the weighted patterns, R_wp_ reliability factor of the patterns, R_exp_—expected weighted profile factor) are summarized in Table 1. The fitting parameters R_p_ and R_wp_ are smaller than 9%, indicating that the obtained refinement results were highly reliable [28]. 

Considering the differences in the ion radii of praseodymium (*r*_Pr_ = 0.9·10^10^ m) and barium (*r*_Ba_ = 1.36·10^10^ m), significant changes in the size of the unit cell parameters could be expected. Despite such significant differences in the size of the ion rays, the admixture of praseodymium ions has an insignificant effect on the size of the unit cell. This fact is connected with the specific structure of layered perovskites; in particular, the presence of bismuth-oxygen layers in their structure prevents drastic changes in the crystal lattice. The results of XRD studies made by Y.Wu and co-authors [29] on BBN ceramics doped by vanadium showed that up to the concentration of additive below 15 atomic% modification has only a slight effect on the unit cell parameters. Above this value of concentration, the process of structural changes is activated due to high stresses in the crystal structure. 

The cell parameters were used to determine the theoretical density of discussed materials. The density was compared with the one obtained by Archimedes’ method—the results are collected in Table 2.

The analysis of the obtained data shows that the addition of Pr^3+^ ions causes a decrease in the density value in relation to the reference composition. 

**Figure 4:**
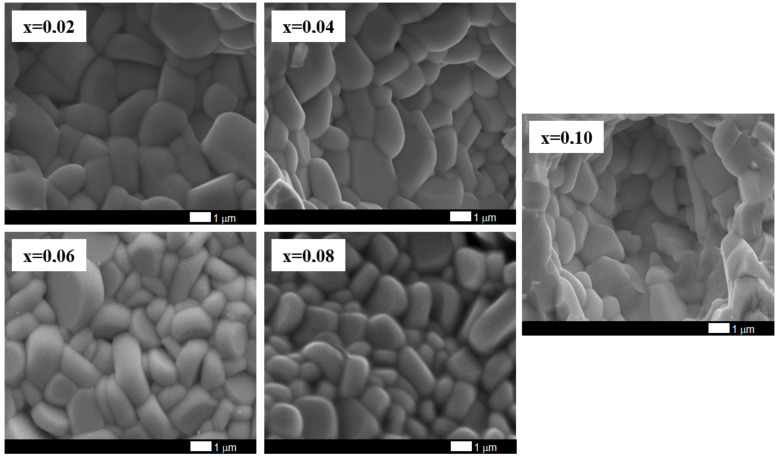
SEM image of BaBi_2_Nb_2_O_9_ ceramics doped with Pr^3+^ ions for different modifier concentrations.

BaBi_2_Nb_2_O_9_ ceramics modified with praseodymium ions were subjected to microstructural examination using a scanning electron microscope. Figure 4 shows the SEM image of BaBi_2_Nb_2_O_9_ ceramics doped with Pr^3+^ ions at a magnification of 10,000×.

The analysis of the obtained images of the ceramic microstructure shows that the increase in the dopant content causes a slight reduction in the grain size. The base material and materials containing praseodymium with a concentration lower than x = 0.06 are characterized by an angular grain shape and emphasized grain boundaries. Starting from the concentration x = 0.06, the grains become rounder and elongate mainly along the Y-axis, assuming a shape much more similar to the plates. Moreover, their growth is layered, which is visible for samples containing Pr^3+^ admixture at concentrations x = 0.08 and x = 0.10. This is a characteristic feature of the morphology of materials belonging to the Aurivillius family in which bismuth layers play a significant role [30]. The presented SEM images of the microstructure of the tested material indicate that the increase in the concentration of Pr^3+^ ions causes a slight grain grinding, which increases their homogeneity. It is also worth noting that with the content of praseodymium ions x = 0.10, there is a change like cracks—for lower dopant content, the cracks are intercrystalline. In contrast, for ceramics containing the highest tested modifier concentration, transcrystalline cracks appear. The average grain size for x = 0 was ca. 2 µm [31], for x = 0.02–1.8 µm, for x = 0.04 was 1.7 µm, for x = 0.06 and x = 0.08 was ca. 1.5 µm, while for x = 0.1 it was ca. 1.4 µm.

The EDS method was used to determine the qualitative and quantitative composition of the chemical elements building BBN ceramics and modified with praseodymium ions. The EDS qualitative analysis confirmed that the obtained results indicate a high homogeneity of the chemical composition of the obtained ceramic materials. The results of the quantitative analysis are presented in Table 3, comparing them simultaneously with the theoretical content of components.

The differences between the obtained values and the theoretical stoichiometry are slight and are within the error limits of the method used. The obtained results indicate a high homogeneity of the chemical composition of the obtained ceramic materials.

**Figure 5:**
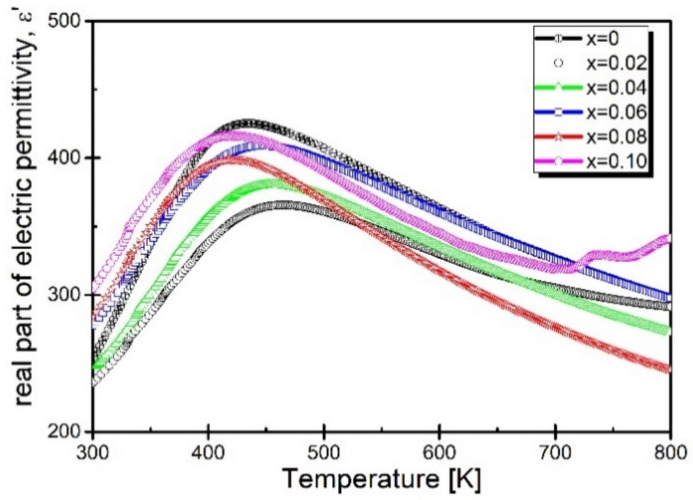
Temperature dependence of the real part of electric permittivity, BaBi_2_Nb_2_O_9_ ceramics modified with Pr^3+^ ions.

The next step in understanding the influence of praseodymium admixture on the properties of BaBi_2_Nb_2_O_9_ ceramics was to subject the samples to temperature measurements of the dependence of dielectric properties. The tests were carried out in a wide range of temperatures, from room temperature to T = 800 K. Figure 5 shows the dependence of the electric permittivity as a function of temperature, BBN ceramics for concentrations x = 0.02–0.10 of praseodymium admixture, measured in the measurement field with the frequency f = 100 kHz.

**Figure 6:**
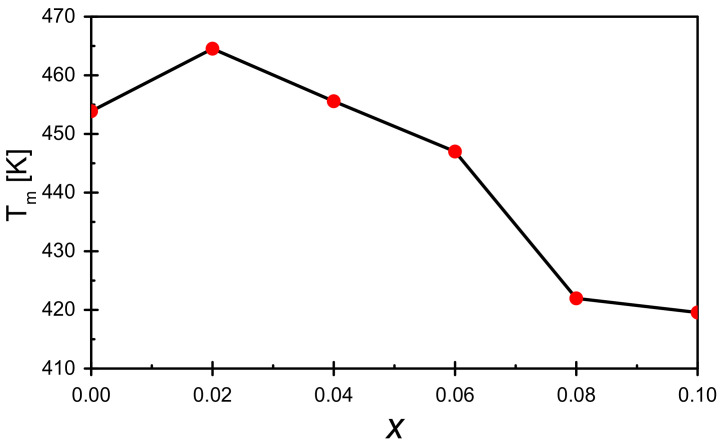
Dependence of the temperature T_m_ determined from the measurement of ε′ (T) on the mole fraction of praseodymium admixture.

The presented temperature dependencies of electric permittivity show that the increasing concentration of praseodymium ions introduced into the sample causes a gradual increase in the value of ε at each temperature ranging from room temperature to T_m_ temperature, corresponding to the maximum dielectric permittivity. It is also worth mentioning that at temperatures close to room temperature, the value of the electric permittivity of each modified sample is higher than the reference sample. The tendency changes in the temperature range slightly lower than T_m_—in this case, the electric permittivity value is higher than recorded for the reference sample only for the concentration x = 0.10. Moreover, what should be noted is a substantial shift of the maximum electric permittivity toward lower temperatures (Figure 6).

**Figure 7:**
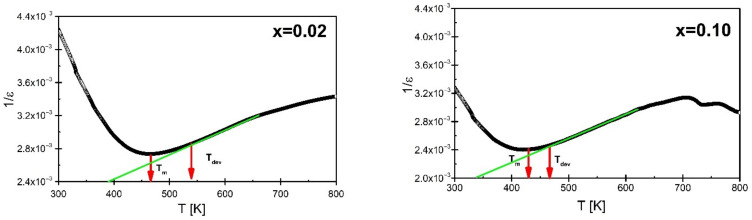
Temperature dependence of the reciprocal of the real component of electric permittivity determined for the heating process, in the measuring field with the frequency f = 100 kHz, for BaBi_2_Nb_2_O_9_ ceramics modified with praseodymium ions for concentrations x = 0.02 and x = 0.10.

The shape of the temperature characteristics of the electric permittivity indicates a strong broadening of the phase transition, which is connected with the inability to apply the Curie–Weiss law in a wide range of temperatures above the T_m_ temperature. The temperature T_dev_, from which the Curie–Weiss law is applied, was determined based on 1/ε′ (T) characteristic (Figure 7). The value of T_dev_ is strongly dependent on praseodymium concentration. Namely, at a concentration of x = 0.02 T_dev_ equals 561 K and gradually decreases with the increase of admixture content. For the BBN ceramics containing x = 0.10 T_dev_ achieves value of 485 K.

In the temperature range where the applicability of the Curie–Weiss law fails, an attempt was made to fit the data to the modified Curie–Weiss law (2) [32]:

(2) $$\frac{1}{\varepsilon^{\prime }}-\frac{1}{{\varepsilon^{\prime }}_{max}}=\frac{{\left(T-T_{m}\right)}^{\gamma }}{C}$$

where:

*ε_max_* is the maximum value of electric permittivity, 

*C*—the Curie-like constant,

*γ*—the diffuseness parameter.

**Figure 8:**
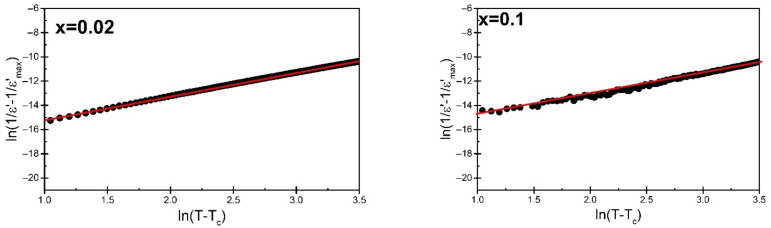
Graph of the dependence of ln (1/*ε* − 1/*ε*_max_) as a function of ln (*T* − *T*_m_)) for BBN ceramics doped with praseodymium in the amount of x = 0.02 and x = 0.1.

An example of such a match for BBN materials doped with praseodymium in amounts x = 0.02 and x = 0.10, respectively, is shown in Figure 8.

**Figure 9:**
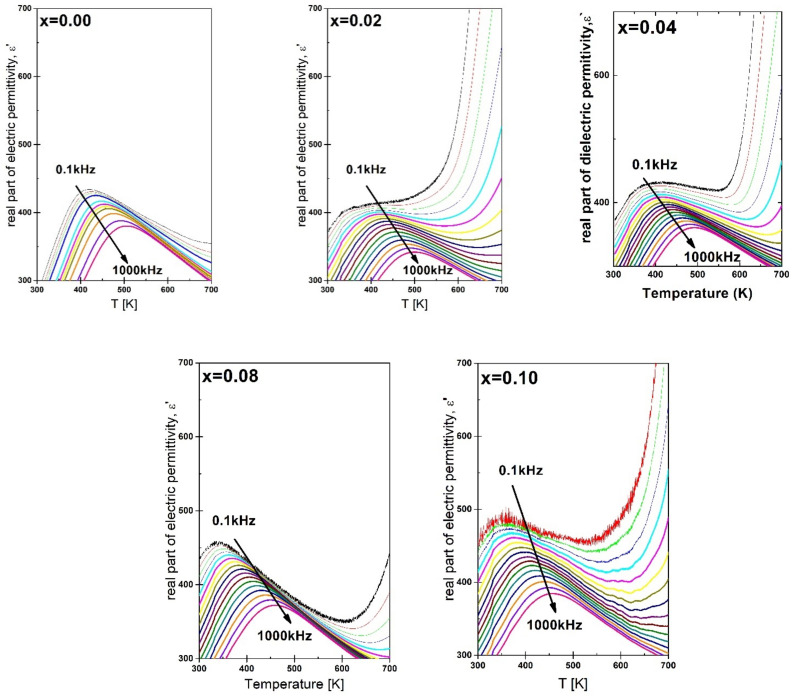
Temperature dependence. The real part of the electric permittivity component of BaBi_2_Nb_2_O_9_ ceramics doped with Pr^3+^ ions for the measurement field in the range f = 0.1 kHz–1 MHz (The arrow in the figures shows the direction of increasing the frequency of the measurement field.).

The discussed fit allowed us to determine the diffuseness parameter γ. In the case of the lowest concentration of praseodymium admixture, a rapid increase in the value of the γ diffusion parameter is observed (Table 4). For the highest amount of modifier, the γ achieved smaller values; however, it never returns to its original value for pure BBN ceramics. The same upward trend in the parameter g was also observed in the case of simultaneous doping of BBN ceramics with Cu and Ta [33] ions [34]. This allows us to speculate that the praseodymium ions, in contrast to vanadium ones [35], enhance the disorder of the crystal lattice [30], which should also influence the frequency dispersion observed in the BBN ceramics. In order to investigate this issue, the temperature dependence of electric permittivity was measured in a wide frequency range of the measurement field. Figure 9 presents the characteristics of the real part of the electric permittivity component ε′ as a function of temperature, carried out for the measurement field in the range f = 0.1 kHz–1 MHz.

The curves presented in Figure 9 show the existence of a substantial frequency dispersion, both the maximum value of the electric permittivity *Δε_max_* and the corresponding temperature Δ*T_m_*. In order to compare the sizes of both dispersions, the degrees of dispersion Δε_max_ (3) and Δ*T_m_* (4) were used, which are defined as follows:

(3) $$\Delta \varepsilon_{max}=\varepsilon_{max100\mathrm{Hz}}-\varepsilon_{max1\mathrm{MHz}}$$

(4) $$\Delta T_{m}=T_{m1\mathrm{MHz}}-T_{m100\mathrm{Hz}}$$

The determined values are summarized in Table 5. The values of Δ*T_m_* differ significantly from those obtained for classic relaxors, such as PMN or PLZT 8/65/35, for which Δ*T_m_* is respectively Δ*T_m_* = 20 K [36] and Δ*T_m_* = 25 K [37]. Their analysis clearly shows that a small admixture of praseodymium ions causes a significant reduction of the discussed frequency dispersion. However, for the content of praseodymium ions at the level of x = 0.08 and x = 0.10, a sharp increase in both parameters is observed again—for the highest discussed concentration, the values of both parameters significantly exceeded the values determined for the reference sample.

**Figure 10:**
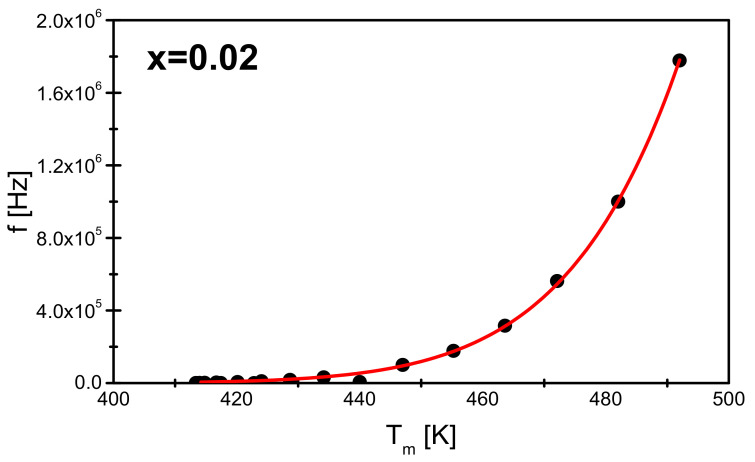
The dependence of *T_m_* on the frequency of the measurement field obtained for the BBN ceramics doped with praseodymium for a mole fraction x = 0.02. The red line marks the fit to the Vogel—Fulcher equation.

The analysis of the results of the ε(T,f) measurements was completed by plotting the characteristics f(*T_m_*) dependences for all discussed ceramic materials. An example of such a characteristic is shown in Figure 10.

The *T_m_*(*f*) relationship was successfully described by the Vogel-Fulcher Equation (5) [5,33,38,39]:

(5) $$f=f_{0}exp\left[\frac{-E_{a}}{k\left(T_{m}-T_{f}\right)}\right]$$

where:

*E_a_*—activation energy related to the mechanism of reorientation of dipole moments,

*T_f_*—freezing temperature of the polarization fluctuation,

*f*_0_—pre-exponential factor.

The fitting procedure allowed to determine the value of the freezing temperature T_f_ and the activation energy *E_a_* for individual samples. The values determined from adjusting the measurement data to the Vogel-Fulcher relationship are summarized in Table 6.

The admixture of praseodymium ions significantly reduces the activation energy associated with the reorientation of the dipole moments while increasing the freezing temperature *T_m_*. Considering the increase in the *T_f_* temperature value and the decrease in the *T_dev_* temperature value, it can be concluded that the temperature range of the occurrence of properties typical for ferroelectric relaxers becomes narrower [33,40].

## 4. Conclusions

The main purpose of the paper was to present the effect of the praseodymium doping on the microstructure, structure, and dielectric properties of BaBi_2_Nb_2_O_9_ ceramics. The applied solid-phase reaction method, followed by the densification of the obtained materials by slow sintering, made it possible to obtain a ceramic material characterized by a tetragonal structure at room temperature with the I4/mmm space group, which was confirmed by X-ray examinations. Based on the analysis of the obtained results, it was found that a small amount of praseodymium did not change the crystal structure of the compound. X-ray phase analysis showed that the produced materials at room temperature are characterized by single-phase grains of the crystalline phase. The analysis of diffraction spectra excludes the presence of foreign phases originating from unreacted compounds. The microstructure analysis carried out with the use of using a scanning electron microscope (SEM) showed that the modification of BaBi_2_Nb_2_O_9_ ceramics with praseodymium ions results in a slight grain refinement, which is characterized by angular, elongated shapes with strongly marked grain boundaries. It was also observed that the cracks formed in the material at lower concentrations are intercrystalline, while those at higher concentrations are transcrystalline.

Modification of the barium niobate compound with praseodymium ions influenced its dielectric properties. Introducing a small concentration of the dopant ion causes a slight increase in the value of electric permittivity and shifts its maximum towards higher temperatures. A further increase in the concentration of modifier cations causes a decrease in the value of electric permittivity. Modifications of the base ceramics lead to a significant increase in the blurring of the maximum of the ε (T) relationship. In all the discussed materials, strong frequency dispersion of the maximum value of the electric permittivity and the corresponding temperature Tm were noted. The Vogel-Fulcher formula describes the relationship f (Tm) in all the discussed samples, which allows the activation energy and freezing temperature to be determined. 

## Figures and Tables

**Table 1:** The lattice parameters, unit cell volume and the reliability factors of BBN ceramics modified into A-subnetwork with Pr^3+^ ions.

Mole Fraction	*a* [Å]	*b* [Å]	*c* [Å]	*V* [Å^3^]	R-Factor [%]
*x* = 0.00	3.9406	3.9406	25.6378	398.1	R_p_ = 6.06 R_wp_ = 8.73 R_exp_ = 4.80
*x* = 0.02	3.9285	3.9285	25.6054	395.2	R_p_ = 6.48 R_wp_ = 8.16 R_exp_ = 4.16
*x* = 0.04	3.9317	3.9317	25.6118	395.9	R_p_ = 6.69 R_wp_ = 8.92 R_exp_ = 4.02
*x* = 0.06	3.9293	3.9293	25.5999	395.2	R_p_ = 6.49 R_wp_ = 8.22 R_exp_ = 4.01
*x* = 0.08	3.9278	3.9278	25.604	395.3	R_p_ = 6.15 R_wp_ = 8.29 R_exp_ = 4.22
*x* = 0.10	3.9316	3.9316	25.604	395.8	R_p_ = 6.73 R_wp_ = 8.16 R_exp_ = 4.27

**Table 2:** Density of BBN ceramics doped in the A-subnetwork with Pr^3+^ ions.

A Mole Fraction	Density *ρ* [g/cm^3^]	+/− (Δ*ρ*) [g/cm^3^]	*ρ/ρ_theoretical_* [%]
0.00	7.071	0.001	97
0.02	6590	0.011	93
0.04	6.866	0.001	97
0.06	6.929	0.004	98
0.08	6.706	0.004	95
0.10	6.758	0.002	96

**Table 3:** Theoretical and experimental statement of the percentage of the elements (expressed as oxides) building ceramics BPBN.

*x*	Theoretical Content [%]				Content of EDS [%]				Error (*σ*^2^) [%]			
	BaO	Bi_2_O_3_	Nb_2_O_5_	Pr_2_O_3_	BaO	Bi_2_O_3_	Nb_2_O_5_	Pr_2_O_3_	BaO	Bi_2_O_3_	Nb_2_O_5_	Pr_2_O_3_
0.02	0.02	21.8	52.6	30	0.02	20.6	51	27.9	1.2	1.6	2.1	0.02
0.04	0.04	21.4	52.6	30	0.04	19.3	51.1	29	2.1	1.5	1	0.04
0.06	0.06	20.9	52.6	30	0.06	18.8	50.6	29.8	2.1	2	0.2	0.06
0.08	0.08	20.5	52.6	30	0.08	19.5	51.4	27.7	1	1.2	2.3	0.08
0.10	0.1	20	52.6	30	0.1	18.7	50	29.4	1.3	2.6	0.6	0.1

**Table 4:** Diffusion parameter γ for different concentrations of praseodymium admixture for BBN ceramics.

Mole Fraction	Parameter *γ*
0.00 [31]	1.45
0.02	1.95
0.04	1.73
0.06	1.73
0.08	1.75
0.10	1.64

**Table 5:** Parameters Δ*T_m_* and Δ*ε_max_* as a function of the admixture of praseodymium ions of the pseudoperovskite structure of BBN ceramics.

Mole Fraction	Δ*T_m_*	Δ*ε_max_*
0.00 [31]	92.59	68
0.02	80.84	61.7
0.04	79.64	56.3
0.06	73.84	72.35
0.08	98.68	78.13
0.10	102	112.1

**Table 6:** List of parameters describing the relaxation properties of BaBi_2_Nb_2_O_9_ ceramics modified with Pr^3+^ cations.

A Mole Fraction	*T_m_* [K] 100 [kHz]	*ε_max_* 100 [kHz]	*E_a_* [eV]	*T_f_* [K]	*f*_o_ [Hz]
0.00 [31]	456	406	0.46	170	9.68·10^12^
0.02	465	366	0.29	267	3.87·10^11^
0.04	456	381	0.26	240	7.12·10^11^
0.06	447	409	0.24	266	1.96·10^11^
0.08	422	399	0.23	246	6.71·10^11^
0.10	420	416	0.19	216	2.56·10^10^

## Data Availability

Not applicable.
